# Polyamines mediate cellular energetics and lipid metabolism through mitochondrial respiration to facilitate virus replication

**DOI:** 10.1371/journal.ppat.1012711

**Published:** 2024-11-18

**Authors:** Yazmin E. Cruz-Pulido, Natalie J. LoMascolo, Delaina May, Jomana Hatahet, Caroline E. Thomas, Andrea K. W. Chu, Samantha P. Stacey, Maria del Mar Villanueva Guzman, Gregory Aubert, Bryan C. Mounce

**Affiliations:** 1 Department of Microbiology and Immunology, Loyola University Chicago, Maywood, Illinois, United States of America; 2 Department of Cellular and Molecular Physiology, Loyola University Chicago, Maywood, Illinois, United States of America; 3 Infectious Disease and Immunology Research Institute, Loyola University Chicago, Maywood, Illinois, United States of America; 4 Division of Cardiology, Department of Internal Medicine, Loyola University Chicago, Maywood, Illinois, United States of America; Heidelberg University, GERMANY

## Abstract

Polyamines are critical cellular components that regulate a variety of processes, including translation, cell cycling, and nucleic acid metabolism. The polyamines, putrescine, spermidine, and spermine, are found abundantly within cells and are positively-charged at physiological pH. Polyamine metabolism is connected to distinct other metabolic pathways, including nucleotide and amino acid metabolism. However, the breadth of the effect of polyamines on cellular metabolism remains to be fully understood. We recently demonstrated a role for polyamines in cholesterol metabolism, and following these studies, we measured the impact of polyamines on global lipid metabolism. We find that lipid droplets increase in number and size with polyamine depletion. We further demonstrate that lipid anabolism is markedly decreased, and lipid accumulation is due to reduced mitochondrial fatty acid oxidation. In fact, mitochondrial structure and function are largely ablated with polyamine depletion. To compensate, cells depleted of polyamines switch from aerobic respiration to glycolysis in a polyamine depletion-mediated Warburg-like effect. Finally, we show that inhibitors of lipid metabolism are broadly antiviral, suggesting that polyamines and lipids are promising antiviral targets. Together, these data demonstrate a novel role for polyamines in mitochondrial function, lipid metabolism, and cellular energetics.

## Author summary

Cells and viruses rely on metabolic circuits to survive and replicate. The small metabolites called polyamines support cellular homeostasis by supporting mitochondrial function. Without polyamines, lipids accumulate in cells because they are not catabolized by fatty acid oxidation. Cells depleted of polyamines switch from aerobic respiration to glycolysis for energy production, similar to cancer cells in hypoxic environments. We also show that varied viruses use lipids to replicate, and targeting lipids with small molecule inhibitors can reduce virus replication. Overall, our work makes a new connection between polyamines and lipid metabolism and highlights potential pathways to target for antiviral therapies.

## Introduction

Polyamines are small, abundant molecules that are positively charged at physiological pH. They play diverse roles in regulating transcription [1–4] and translation [5–7], cellular signaling events [8,9], and cell cycling [10,11], among others [12]. Cellular polyamine synthesis is tightly regulated at the levels of synthesis, breakdown, and interconversion [13,14], and cells expend significant energy to maintain polyamine pools. Interestingly, however, polyamines can be depleted from cells via specific inhibitors, including difluoromethylornithine, or DFMO, which irreversibly inhibits ornithine decarboxylase 1 (ODC1), the first step in polyamine synthesis [15,16]. DFMO treatment of cells *in vitro* and animals *in vivo*, including humans, is generally well-tolerated, with few, reversible side effects [17]. Thus, despite their importance in a variety of critical cellular processes, polyamine depletion does not appear to grossly affect cellular health and function. Importantly, however, polyamine depletion leads to changes in cellular transcription [18,19], translation [7], and metabolism [20,21]. In particular, the polyamine spermidine contributes to mRNA translation through the unique modification on eIF4G called hypusination. Spermidine is conjugated to eIF4G and hydroxylated to form hypusinated eIF4G (hyp-eIF4G), which facilitates in the translation of a subset of cellular mRNAs. Several polypeptide motifs have been shown to require hyp-eIF4G, particularly poly-proline motifs. Inhibitors of hypusination like GC7, ciclopirox, and deferiprone have shown antiviral efficacy against coronaviruses [22] and Ebolavirus [23] *in vitro*, and there is significant potential to treat viral infection with inhibitors targeting hypusination.

Polyamine depletion can lead to changes in cellular metabolism. We recently showed [18] that polyamine depletion reduces translation of sterol regulatory element binding protein 2 (SREBP2) mRNA, a master transcription regulator of cholesterol synthesis genes. This results in decreased cellular cholesterol, which had previously been described in rats and mice treated with DFMO [24,25]. Others have shown that polyamines modulate nucleotide metabolism [26] and interact directly with nucleotides [27]; conversely, we have shown [28] that nucleotide metabolism can impact polyamine levels. The global effects of polyamine depletion remain to be fully explored, though it is clear that polyamines are essential regulators of cellular homeostasis.

Lipids are key components of cells, and their synthesis and regulation are key to cellular homeostasis. Lipids play several roles within the cell, including as components of cellular membranes, as critical signaling molecules, and as a significant source of cellular energy. Polyamines have been described to impact lipid metabolism, specifically by enhancing lipolysis [29] and protecting lipids from peroxidation [30,31]. Additionally, lipids impact the polyamine transporter ATP13A2 to facilitate polyamine transport into cells [32,33]. Precisely how polyamines impact lipolysis and whether they affect other stages in lipid synthesis or breakdown remains to be fully understood.

Viruses use both lipids and polyamines to facilitate productive infection. We and others have shown that diverse viruses rely on polyamines for multiple steps in the viral lifecycle, including attachment and entry [18,22,34], translation [23,35,36], transcription and replication [36–38], and egress and packaging [39,40]. This has been demonstrated for a wide variety of viruses, including RNA and DNA viruses of distinct classes [41]. Similarly, lipids are involved at nearly every stage of virus replication, from mediating membrane fusion in enveloped viruses to serving as a scaffold for nucleic acid synthesis once the virus has entered and begun replication. Lipids are also key signaling molecules in antiviral interferon responses [42], as well as in inflammatory responses [43]. Molecules targeting lipid synthesis show promise as antiviral agents, as do molecules that target polyamines, like DFMO.

Given the link between viruses and both polyamines and lipids, we gained an interest in a potential connection between lipid and polyamine metabolism. We observed that cells depleted of polyamines have increased lipid droplet content. Curiously, lipid synthesis genes were downregulated with polyamine depletion. To reconcile this disparity, we measured lipid oxidation, finding lipid catabolism is markedly decreased with polyamine depletion. We further discovered that polyamines mediate mitochondrial structure and function, and cells depleted of polyamines derive their energy primarily from glycolysis, in a polyamine-mediated Warburg-like effect. These data highlight a novel connection between polyamines, mitochondrial respiration, and lipid synthesis and also highlights both polyamines and lipid metabolism as potential antiviral targets.

## Results

### Lipid droplets accumulate in polyamine-depleted cells

**Fig 1:**
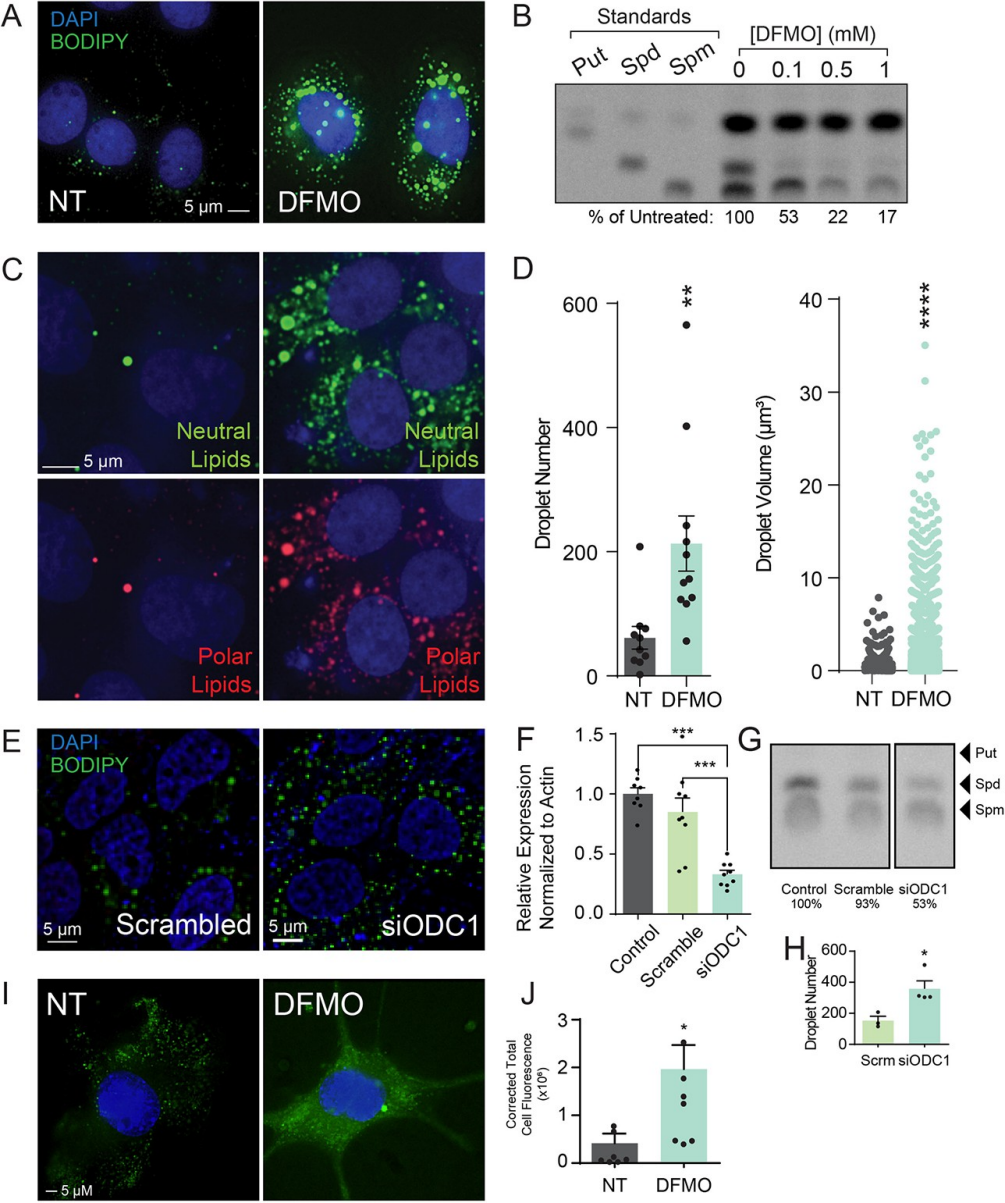
Lipid droplets accumulate in polyamine-depleted cells. Huh7 cells were treated with 1 mM DFMO for four days and (A) visualized for lipid droplets by staining for BODIPY (green) and nuclei by DAPI (blue). (B) Polyamine depletion was confirmed in cells treated with increasing doses of DFMO for four days by thin layer chromatography. (C) Huh7 cells were treated as in (A) but stained with Nile Red to differentiate neutral/nonpolar lipids (green) from polar lipids (red). (D) Lipid droplets from (A) were quantified for droplet number per cells and droplet volume. (E) siRNA targeting ODC1 (“siODC1”) or a scrambled siRNA (“scrambled”)were transfected into Huh7 cells and lipid droplets visualized four days later as in (A). Knockdown of ODC1 was verified by (F) qPCR for ODC1 mRNA expression, comparing control transfected cells to scrambled and ODC1-specific siRNA, and (G) TLC of polyamines in transfected cells. Values listed below indicate the relative quantity of total polyamines in the sample normalized to the control cells, as measured in ImageJ. (H) Quantitation of the number of lipid droplets within scramble siRNA and siODC1 samples from (E) as measured in ImageJ. (I) Murine embryonic fibroblasts (MEFs) were treated with 1 mM DFMO for four days prior to visualizing lipid droplets as in (A). (J) Quantitation of corrected total cell fluorescence in MEFs from (I) as measured in ImageJ. Bars indicate 5 μm in immunofluorescent images. **p<0.01; ***p<0.001; ****p<0.0001 by Student’s T-test, n≥3.

As an initial consideration of lipid homeostasis in cells, we treated Huh7 hepatocarcinoma cells with DFMO for four days to mediate polyamine depletion and then imaged these cells after staining with the lipid stain Bodipy 493/503, which stains for neutral lipids, including lipid droplets. We observed that with DFMO treatment, lipid droplets grew in both size and number (Fig 1A). We confirmed that DFMO mediated polyamine depletion by thin layer chromatography (TLC) of dansylated polyamines (Fig 1B). We further used Nile Red (9-(Diethylamino)-5H-benzo [a]phenoxazin-5-one), which differentially stains lipids, fluorescing green for nonpolar and red for polar lipids [44]. As with our Bodipy 493/503 stain, we observed larger and more numerous polar and nonpolar lipid droplets with DFMO treatment (Fig 1C). With our images of Bodipy stained cells, we quantified lipid droplet number and volume and observed a significant increase in both with DFMO treatment (Fig 1D). We confirmed these results genetically by knocking down ODC1, the gatekeeper enzyme for polyamine synthesis, via siRNA. Upon knockdown, we observed phenotypes similar to those of DFMO treatment (Fig 1E), suggesting that the lipid accumulation was not an artifact of the small molecule inhibitor. We confirmed siRNA-mediated knockdown via qPCR (Fig 1F) and TLC (Fig 1G). We counted the number of lipid droplets in the scrambled siRNA and siODC1 cells using ImageJ and observed a 2.4-fold increase in droplet number (Fig 1H). We also performed immunofluorescence in murine embryonic fibroblasts (MEFs), primary fibroblast cells, to determine if the observed phenotype was generalizable beyond Huh7 cells. We found that MEFs exhibited more diffuse lipid droplet staining and that this signal increased with DFMO treatment (Fig 1I, quantified in 1J via ImageJ). Overall, our results demonstrate that lipid homeostasis is dysregulated with polyamine depletion in Huh7 cells.

### Expression of lipid biosynthetic genes is decreased with polyamine depletion

**Fig 2:**
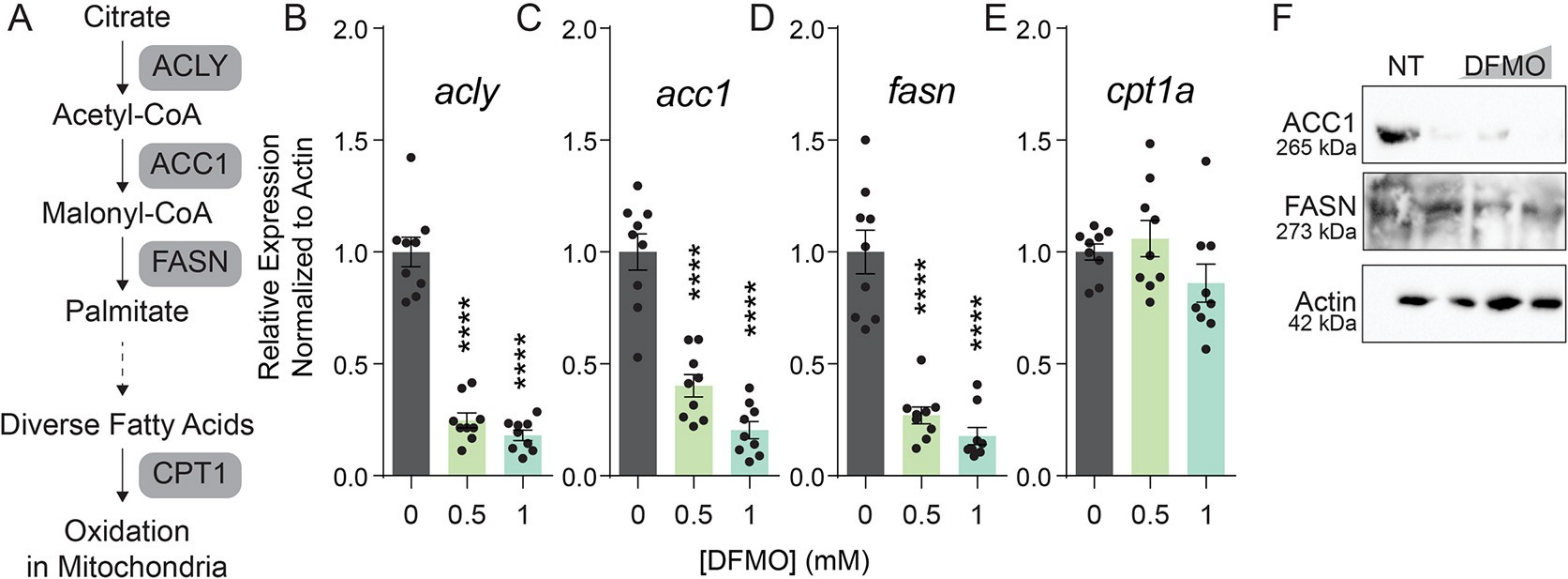
Expression of lipid biosynthetic genes is decreased with polyamine depletion. (A) Diagram of the initial steps in lipid synthesis. Lipids and their products are centered, and the enzymes participating in lipid metabolism are highlighted in gray. (B-F) Huh7 cells were treated with DFMO at 0.5 or 1 mM for four days prior to collection for qPCR (B-E) and cells were treated with 0.1, 0.5, and 1 mM DFMO prior to collection for western blot (F) for the genes and proteins indicated, as highlighted in (A). qPCR data were normalized to β-actin and untreated controls to calculate relative gene expression. ****p<0.0001 by Student’s T-test, n = 3.

Given that we see accumulation of lipid droplets in DFMO treated cells, we anticipated that expression of lipid synthetic enzymes may be upregulated in these cells. Previously [18], we had measured the changes to the transcriptome upon polyamine depletion via DFMO, and we had observed changes in lipid metabolism genes. To verify these transcriptomic data, we designed primers against a subset of genes within the lipid synthesis pathway (Fig 2A) for *acly*, the enzyme that converts citrate to acetyl-CoA (Fig 2B); *acc1*, encoding the enzyme that converts acetyl-CoA to malonyl-CoA (Fig 2C); *fasn*, which encodes fatty acid synthase (Fig 2D); and *cpt1a*, which encodes carnitine palmitoyltransferase 1A involved in mitochondrial transport of fatty acids for fatty acid oxidation (Fig 2E). Given that DFMO treatment results in the accumulation of lipid droplets, we anticipated that the expression of these genes (or a subset of these genes) may be upregulated. Interestingly, we observed that DFMO treatment resulted in a significant decrease in gene expression of *acly*, *acc1*, and *fasn*, in stark contrast to our expected results. We confirmed by western blot that both ACC1 and FASN were reduced in abundance (Fig 2F), suggesting that polyamine depletion leads to a decrease in the transcription and translation of these enzymes’ mRNA, though lipid droplets accumulate within treated cells.

### SREBP1 requires polyamines for translation

**Fig 3:**
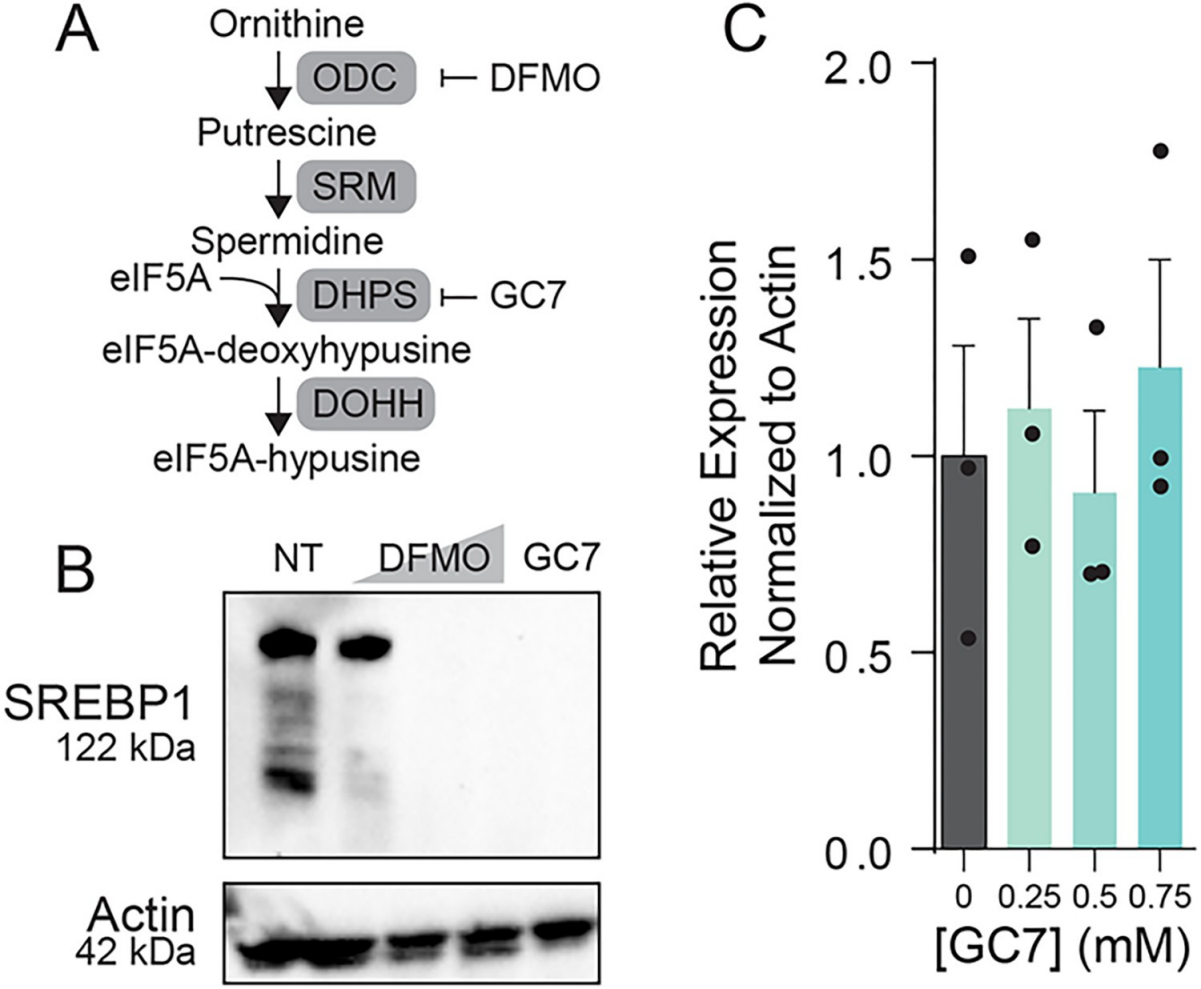
SREBF1 requires polyamines for translation. (A) Diagram of polyamine synthesis, hypusination, and inhibitors of the pathway. Pertinent enzymes are highlighted in gray. GC7 and DFMO inhibit hypusination and polyamine synthesis by inhibiting ODC and DHPS, respectively. (B) Huh7 cells were treated with 0.1, 0.5, and 1 mM DFMO for four days or 500 μM GC7 for 24h prior to collection of samples for western blot, probing for SREBP1. (C) Cells were treated with increasing doses of GC7 and transcription of *srebf1* measured by qPCR using specific primers and normalizing to β-actin and untreated controls to calculate relative gene expression (n = 2). Western blot is representative of n≥3.

Given that we observe more than one lipid synthesis gene decreased by polyamine depletion, we considered whether higher-level regulatory mechanisms may be at play. ACLY, ACC, and FASN are transcriptionally regulated by the protein sterol regulatory element binding protein 1 (SREBP1, also known as sterol regulatory element-binding transcription factor 1 [SREBF1]), which coordinates transcription of a variety of lipid synthesis genes. We previously showed [18] that SREBP2, the master regulator of cholesterol synthesis, was sensitive to polyamine depletion at the level of protein synthesis. Namely, SREBP2 transcription was unchanged, but its translation was significantly reduced. This was due to the role of polyamines in a process called hypusination (Fig 3A). Spermidine is conjugated to eIF5A and hydroxylated to form a modified amino acid called hypusine. This hypusination of eIF5A is essential in translating a subset of cellular proteins, including SREBP2. To determine if SREBP1 is regulated by polyamines, specifically through hypusination, we measured its abundance by western blot following DFMO treatment (Fig 3B). We observed reduced SREBP1 protein with DFMO treatment, so to test if this reduction was due to hypusination of eIF5A, we used the specific inhibitor GC7, which targets the enzyme deoxyhypusine synthase to block eIF5A-hypusine, in contrast to DFMO, which globally blocks polyamine synthesis. By western blot, we observed a decrease in protein expression with GC7 treatment, similar to DFMO treatment. In fact, with GC7 treatment, we observed detectable no SREBF1 protein. We checked if *srebf1* transcript levels were decreased with GC7 treatment through qPCR, and we observed no significant change in transcript levels (Fig 3C), suggesting that SREBP1 is primarily regulated by polyamines at the level of translation, likely through hypusination of eIF5A.

### Polyamine depletion reroutes energy production to glycolysis

**Fig 4:**
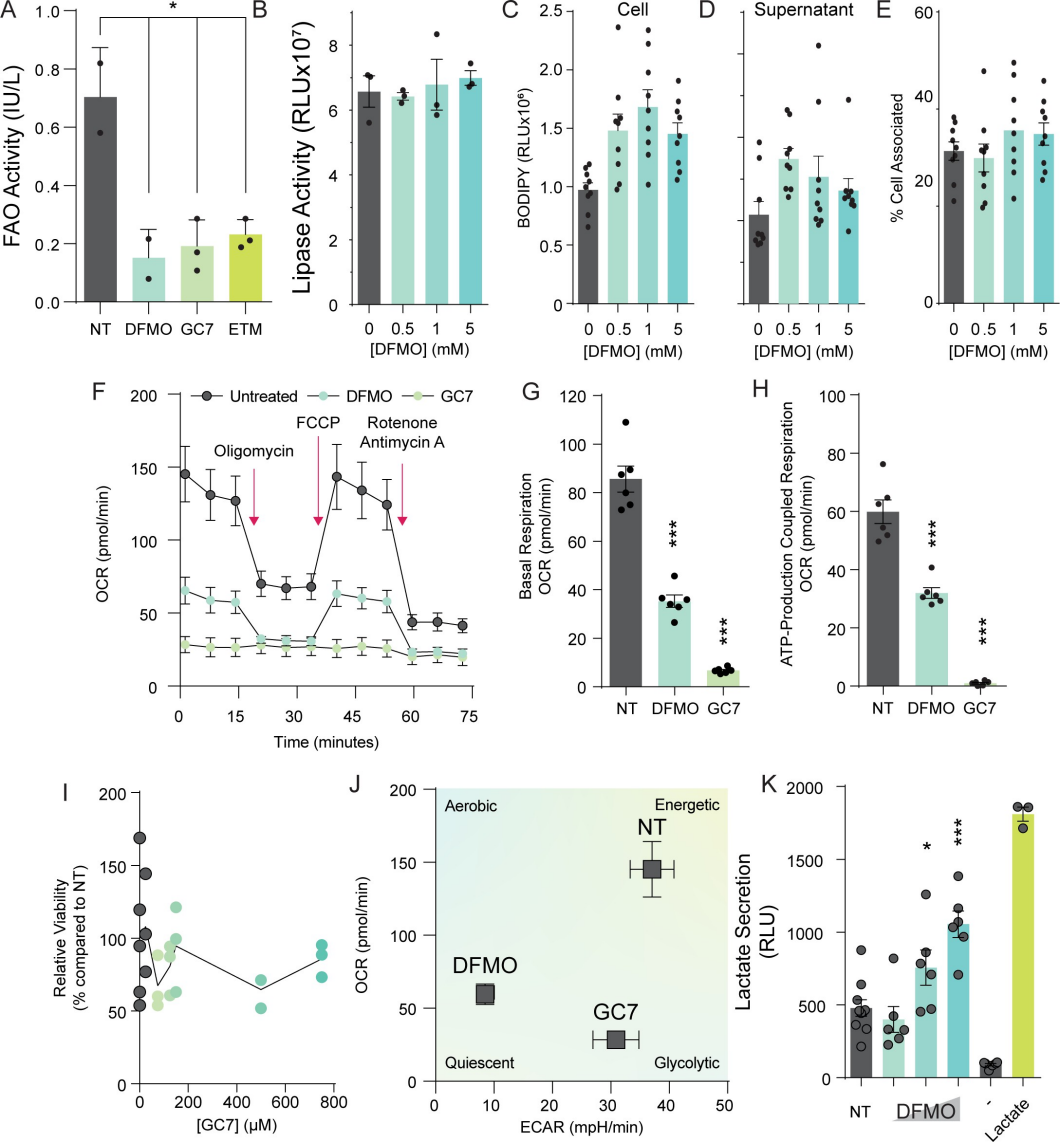
Polyamine depletion reroutes energy production to glycolysis. (A) Huh7 cells were treated with 1 mM DFMO for four days, 500 μM GC7 for 24h or 40 μM etomoxir (ETM) for 24h, and fatty acid oxidation (FAO) activity was measured by fluorescent assay. Standard curves were used to quantify activity. (B) Huh7 cells were treated with increasing doses of DFMO (0.5, 1, and 5 mM) as indicated for four days prior to being assayed for cellular lipase activity by commercial kit. Raw relative luciferase values were measured. (C) Huh7 cells were treated with increasing doses of DFMO (0.5, 1, and 5 mM) as indicated for four days prior to measuring lipid uptake by cells by measuring incorporation of BODIPY from the supernatant into cells. Briefly, cells were incubated with BODIPY for 15m to allow for lipid uptake. Cells were then washed and media replenished. Fluorescent BODIPY levels in the cell were measured 24h later via fluorescent plate reader. (D) Supernatant from cells described in (C) was assayed for BODIPY levels to measure secretion of BODIPY after 24h incubation. (E) The ratio of cell-associated (C) to extracellular (D) BODIPY signal was measured by dividing the cell-associated fluorescence from BODIPY by the extracellular BODIPY fluorescence. (F) Huh7 cells were treated with 1 mM DFMO for four days or 500 μM GC7 for 16h and oxygen consumption rate (OCR) was measured by Seahorse XF Cell Mito Stress Test. Red arrows indicate the time of addition of inhibitors of the mitochondrial electron transport chain to measure basal, maximal, and minimum oxygen consumption through the electron transport chain. (G) Basal respiration and (H) ATP production-coupled respiration were derived from data in (B). (I) Huh7 cells were treated with increasing doses of GC7 for 16h and cell viability measured by MTT assay. Values were normalized to untreated controls. (J) Metabolic state was derived from data in (F) to differentiate between the impact of DFMO and GC7 on Huh7 energetics. (K) Glycolytic activity was measured by Lactate-GLO assay after treatment with increasing doses of DFMO (0.5, 1, and 5 mM) for four days prior to assay. “-”indicates blank control; “Lactate” indicates sample spiked with lactate. Values represent raw luciferase values measured. *p<0.05; ***p<0.001 by Student’s T-test, n = 2 (A-D; F, G-K) or n = 3 (E).

We observed a decrease in the expression of lipid synthetic genes, including the translation of the major transcription factor SREBP1. However, these results do not align with our initial observation of increased lipid droplet size and number. One possibility is that accumulation of lipids results in a negative feedback loop that reduces lipid synthesis gene expression and translation. Lipid homeostasis is the result of a balance between synthesis and breakdown, which occurs in the mitochondria via fatty acid oxidation. To investigate whether lipid oxidation might be modified with polyamine depletion, we first measured lipid oxidation via a fluorescent reporter kit. We observed a significant decrease in lipid oxidation in cells treated with either DFMO or GC7 (Fig 4A), similar to treatment with our positive control etomoxir, suggesting that both inhibition of polyamine synthesis and hypusination contributes to lipid oxidation. Lipid flux also depends on the breakdown of cellular lipids, facilitated by lipases. To determine if polyamine depletion might have an effect on lipase activity, we measured global cellular lipase activity using a commercially available kit. Upon treating cells with increasing doses of DFMO to deplete polyamines, we noted no significant change in cell-associated lipase activity (Fig 4B). We further considered that polyamine depletion could affect lipid uptake into the cells, resulting in changes in lipid droplet size. To test this, we added BODIPY, a fluorescent lipid, to the media of cells treated with DFMO to deplete polyamines. We incubated the BODIPY with the cells for 15 minutes and then removed the media and replenished with fresh BODIPY-free media. Finally, we measured BODIPY levels 24h later both intracellularly (Fig 4C) and extracellularly (Fig 4D), as well as the ratio (Fig 4E). We found that BODIPY uptake was increased in cells treated with DFMO, and we also observed that this uptick in BODIPY signal was reflected in the extracellular space after 24h, resulting in a ratio of signal that was unchanged with DFMO treatment. Together, these data suggest that lipid uptake is moderately increased upon polyamine depletion and that these lipids become part of the homeostatic lipid flux within the cells. Finally, we considered whether the mitochondria of the Huh7 cells functioned in cellular energetics to alter lipid metabolism, and we measured cellular oxygen consumption by Seahorse XF Cell Mito Stress Test Kit. In these assays, we can measure acidification of the cellular media as a readout of cellular energetics, specifically the oxygen consumption rate (OCR). The cells are then stressed with inhibitors of the mitochondrial oxidative phosphorylation pathway. Treatment with oligomycin inhibits ATP synthesis and reduces OCR; FCCP collapses the proton gradient resulting in maximum oxidative phosphorylation and respiration. Rotenone combined with antimycin results in complete inhibition of oxidative phosphorylation and a minimum level for OCR. We found that the OCR in Huh7 cells was sensitive to oligomycin (ATP synthase inhibitor), FCCP (carbonyl cyanide-p-trifluoromethoxyphenylhydrazone; uncoupler of mitochondrial oxidative phosphorylation), and rotenone and antimycin A (inhibitor of complexes I and III of mitochondrial respiration) (Fig 4F). The cells responded to each of the inhibitors as expected, with oligomycin dropping OCR, FCCP maximizing, and rotenone/antimycin reducing OCR to its minimum. Interestingly, cells treated with DFMO or GC7 exhibited low cellular respiration as measured by the OCR at a basal level, and their responses to the inhibitors were muted in comparison to untreated cells. When analyzed further, these cells were found to have reduced basal respiration (Fig 4G) and ATP-production coupled respiration (Fig 4H). We observed no decrease in cellular viability (Fig 4I) Further, we find that cells treated with DFMO exhibited characteristics of quiescent cells, though GC7-treated cells exhibited characteristics of glycolytic cells (Fig 4J). Overall, our data suggest that decreased polyamine levels and hypusination result in decreases in mitochondrial function, including lipid oxidation. However, cells are still alive despite dramatic changes to their respiration. Thus, we considered that the cells may have switched from mitochondrial respiration to glycolysis for energy production, in a Warburg-like effect. To measure glycolysis, we used the Lactate-GLO fluorescent assay, which measures lactate production as a product of glycolysis. In cells treated with DFMO, we observed a significant increase in lactate production compared to untreated cells (Fig 4K), suggesting that glycolysis is upregulated in polyamine-depleted cells.

### Mitochondrial structure is compromised by polyamine depletion

**Fig 5:**
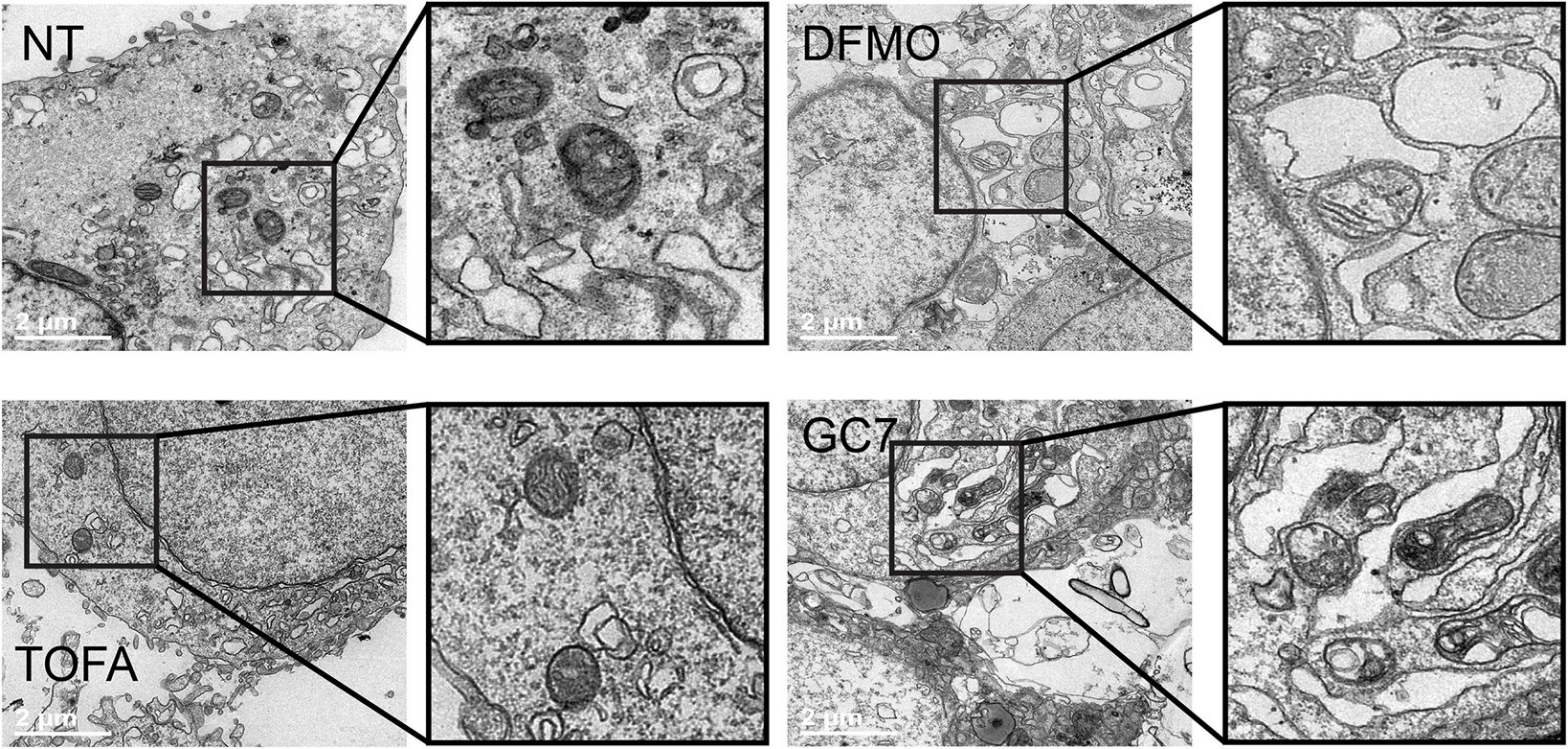
Mitochondrial structure is compromised by polyamine depletion. Huh7 cells were treated with 1 mM DFMO, 2 μg/mL TOFA, or 500 μM GC7 and cellular structures were analyzed by electron microscopy. Bar indicates 2 μm. Black outlines indicate zoomed areas to highlight detail in mitochondrial structure.

Given the central role of the mitochondria in cellular respiration and lipid oxidation for energy production, we next considered if mitochondrial structure might be impacted by DFMO or GC7 treatment. We imaged Huh7 cells treated with either inhibitor by electron microscopy, observing electron-dense mitochondria in untreated cells. In DFMO- and GC7-treated cells, however, we found that mitochondria were misformed, with poorly defined cristae and a loss of electron density (Fig 5). With TOFA treatment, we observed no change in mitochondrial morphology, though the molecule inhibits lipid synthesis. Overall, these data suggest that mitochondrial structure and function is compromised in cells depleted of polyamines.

### Virus infection relies on lipid production

**Fig 6:**
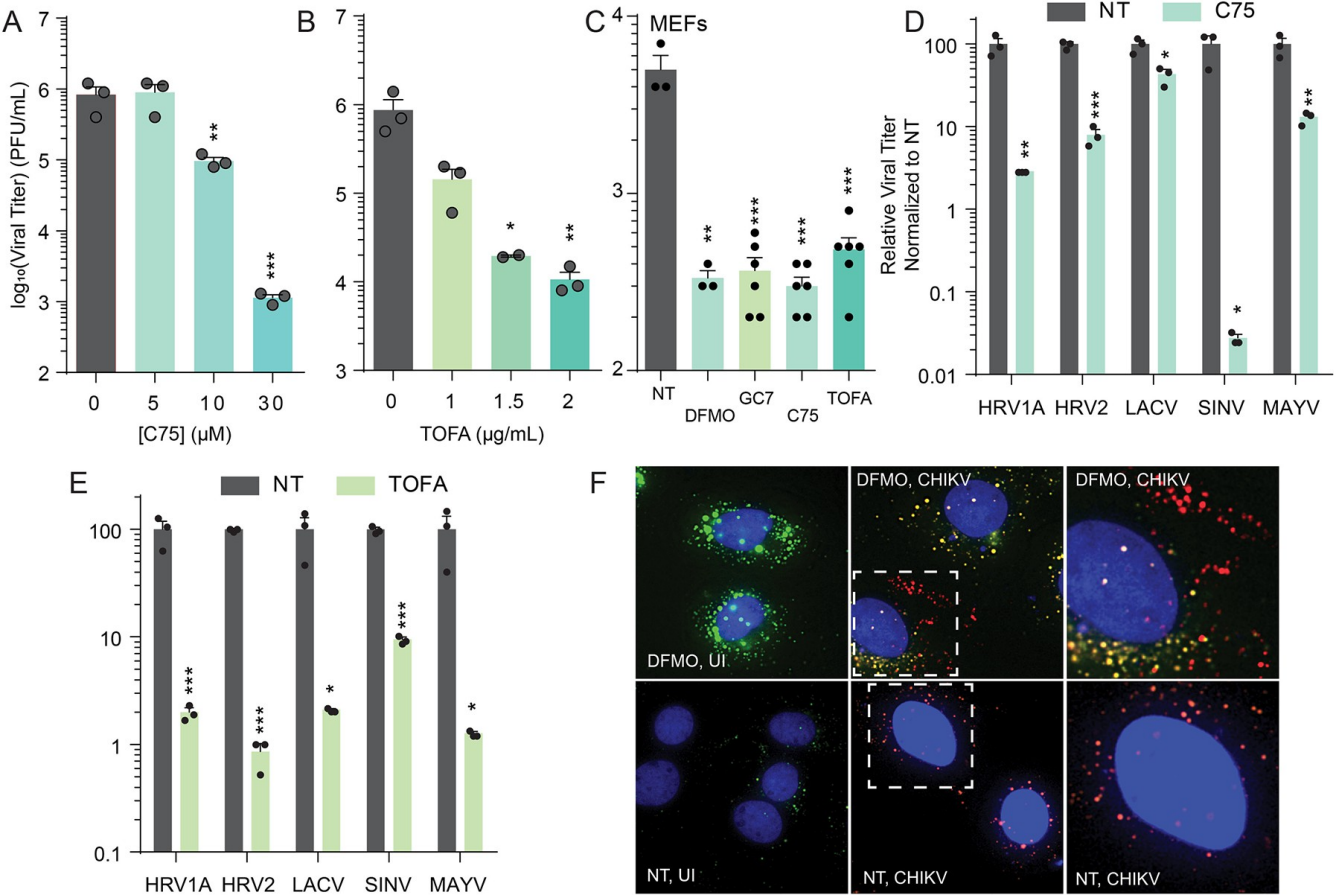
Virus infection relies on lipid production. Huh7 cells were treated with increasing doses of (A) C75 (5, 10, and 30 μM), a FASN inhibitor, and (B) TOFA (1, 1.5, and 2 μg/mL), an ACC1 inhibitor, and subsequently infected with chikungunya virus (CHIKV) at multiplicity of infection (MOI) of 0.1 PFU/cell for 24h. Viral titers were determined by plaque assay on Vero cells. (C) Murine embryonic fibroblasts (MEFs) were treated with 500 μM DFMO for four days, 500 μM GC7 for 16h, 100 μM C75, or 2 μg/mL TOFA, as indicated, infected with CHIKV at MOI 0.1, and viral titers determined at 24 hpi by plaque assay on Vero cells. Cells were treated with (D) 100 μM C75 or (E) 2 μg/mL TOFA and infected with human rhinovirus 1A (HRV1A), human rhinovirus 2 (HRV2), La Crosse virus (LACV), Sindbis virus (SINV), or Mayaro virus (MAYV). Titers were determined at 24h by plaque assay. (F) Huh7 cells were treated with 1 mM DFMO for 4 days and subsequently infected with CHIKV at MOI 1 for 24h. Cells were fixed and stained for dsRNA (red; viral replication compartments) and lipid droplets (green, BODIPY). Overlap of dsRNA and lipid droplets is shown through orange/yellow staining. Boxed image is magnified to the right of the images. *p<0.05; **p<0.01; ***p<0.001 by Student’s T test, n≥3.

Viruses are widely described to use lipids at several stages in their replication, including entry, replication, and egress. Cytoplasmic viruses frequently replicate in membranes and, thus, rely on cellular lipid synthesis for efficient replication. Using chikungunya virus (CHIKV) as a model, we treated cells with increasing doses of C75 (Fig 6A) and TOFA (Fig 6B). C75 reduces lipid synthesis by inhibiting fatty acid synthase (FASN), and TOFA similarly reduces lipid synthesis by inhibiting acetyl-CoA carboxylase (ACC1). After a short pretreatment, we infected cells at a multiplicity of infection (MOI) of 0.1 and then measured viral titers by plaque assay at 24 h post infection (hpi). We observed a significant dose-dependent decrease in viral titers with both inhibitors. To determine if these inhibitors were antiviral in another cellular system, we performed similar analyses in murine embryonic fibroblasts (MEFs). We treated with DFMO (500 μM), GC7 (500 μM), C75 (100 μM) and TOFA (2 μg/mL), infected and measured viral titers by plaque assay, as we had performed in Huh7 cells. We observed that each of these inhibitors was antiviral (Fig 6C). We extended our investigation of C75 and TOFA to several other viruses (Fig 6D for C75 and Fig 6E for TOFA), including human rhinoviruses 1A and 2 (HRV1A and HRV2; enteroviruses), La Crosse virus (LACV, a bunyavirus), and Sindbis and Mayaro viruses (SINV and MAYV, alphaviruses). We observed that both C75 and TOFA inhibited virus replication, to varying extents. SINV appeared dramatically sensitive to C75, while LACV was largely resistant. All viruses exhibited at least a 10-fold reduction with TOFA.

As mentioned, cytoplasmic virus replication frequently occurs on membranes, derived from the host cells, and used as sites of RNA synthesis, termed replication factories. To visualize these factories, we infected DFMO-treated, polyamine-depleted cells with CHIKV and visualized replication compartments during infection by staining for dsRNA, a hallmark of virus infection. As expected, in uninfected samples, we observe some lipid droplets in not treated, uninfected cells (“NT, UI”), while DFMO-treated uninfected cells (DFMO, UI) have more stain for lipid droplets. The stain for dsRNA and lipid droplet overlaps in untreated Huh7 cells (“NT, CHIKV”), shown more clearly in the zoomed-in image, showing overlap of red and green to make yellow. However, in DFMO-treated and infected cells (“DFMO, CHIKV”), we observed replication compartments that did not overlap with lipid stain (red signal, especially apparent in the zoomed-in image). These data suggest that, despite increased lipid droplet accumulation with polyamine depletion, these lipid droplets may not be compatible with processes relevant to virus replication.

## Discussion

Polyamines play various roles in the cell, which has been extensively reviewed [45–49], though we continue to learn new mechanisms by which these molecules facilitate basal cellular processes. Here, we show that polyamines promote lipid homeostasis by maintaining cellular oxidative phosphorylation through proper mitochondrial structure and function. Precisely how polyamines aid in mitochondrial function isn’t clear, though. We observe that cells depleted of polyamines, or in which hypusination is inhibited, mitochondrial structure is disrupted, which compromises mitochondrial function, specifically lipid oxidation. This change in mitochondrial structure could be a result of structural proteins not expressed or properly localized, for instance. With this significant compromise in the cell’s ability to produce energy, we find that these cells shift to glycolysis, in a Warburg-like effect. We have previously shown [18,36] that cells treated with DFMO or GC7 exhibit no change in cellular viability, and one mechanism by which these cells may survive without polyamines and without functional mitochondria may be this glycolytic shift.

Polyamine-depleted cells are viable, and cells can tolerate large doses of DFMO. In the case of viral infection, polyamine-depleted cells have reduced titers that are fully rescued upon supplementation of cells with exogenous polyamines. While polyamines are critical for cellular functions, including mitochondrial function as shown here, they are dispensable for cellular homeostasis, as depleting cells with the specific inhibitor difluoromethylornithine (DFMO) reduces proliferation but has no detectable impact on cell viability [50]. DFMO is used clinically in the treatment of trypanosomiasis, and humans tolerate large, chronic doses of the drug with minor (and reversible) side effects [51,52]. Thus, polyamines are key resources, important for viral replication, but cells and organisms tolerate polyamine depletion. As such, targeting polyamines as an antiviral strategy holds significant promise, though there are hurdles, including the need for long-term treatment and the potential for antiviral resistance.

In addition to the impact of polyamines on mitochondrial structure and function, we also noted that polyamines promote lipid synthesis through the translation of SREBP1 mRNA, specifically via hypusination of eIF5A. Previously, we showed that SREBP2, the key transcriptional regulator of cholesterol synthesis, is also sensitive to polyamine levels through hypusination [18], and these new findings suggest that sterol regulatory element transcriptional regulators are more broadly sensitive to polyamine levels. Though the precise mechanisms by which hypusination facilitates translation remain to be fully worked out, the consensus is that hypusinated eIF5A promotes the translation of “hard-to-translate” regions, such as poly-proline tracts [6,53,54]. SREBP1 (as well as SREBP2) has several diproline motifs, and hypusinated eIF5A may promote mRNA translation through these motifs. Inhibiting this process could slow the rate of translation, resulting in lower basal levels of SREBP1, as we have observed.

Our and others’ prior work has demonstrated a variety of functions for polyamines in virus infection [45,45,55]. In the case of CHIKV, polyamines facilitate viral genome replication and translation [36]. In fact, when CHIKV is passaged in the presence of DFMO, the observed mutations promote the expression of the viral RNA-dependent RNA polymerase [56]. Additionally, we observe mutations in nsP1, including in a membrane-binding domain [56]. At the time, we did not fully understand the function of this mutation, though it did confer enhanced membrane attachment. In light of the findings in this manuscript, this nsP1 mutation may emerge as a result of altered lipids in cells due to changes in lipid oxidation. Identification of the precise lipids changed with polyamine depletion, as well as which lipids function to facilitate CHIKV replication, will further inform the function and mechanism behind this mutation. Regardless, the ability of CHIKV to overcome polyamine depletion by mutation of the membrane-binding domain of nsP1 aligns well with our current findings reported here.

Lipids have a variety of functions in virus infection, from entry, to replication, to egress. Lipids are also central to the structure of enveloped viruses. As such, the ability of viruses to use and manipulate lipids, their abundance, localization, and metabolism is key to successful replication. The vast majority of viruses replicate their genomes in host-derived membranes, in viral replication factories, and distinct viral families derive these lipids from specific cellular compartments. Disruption of either cellular lipids or the viruses’ ability to manipulate cellular lipids, can inhibit virus replication and is a tantalizing drug target. However, manipulating cellular lipids to disrupt viral process without harming cellular homeostasis requires consideration.

As mentioned, polyamines are critical to cellular function, and the cell expends significant energy in maintaining appropriate levels of polyamines to facilitate these processes. However, cells and organisms survive without their polyamines, which at face-value appears incongruous with their myriad roles. However, our work here demonstrates that cells have evolved mechanisms to survive without polyamines, specifically in this case by shifting from oxidative phosphorylation to glycolysis for energy production. Additional compensatory mechanisms, such as using alternative counterions or molecules, may allow for cells and organisms to survive, perhaps indefinitely, in environments lacking polyamines. Such observations highlight the potential for targeting polyamines as a therapeutic, including as an antiviral. Depleting polyamines has the potential to significantly reduce viral replication, which has been described *in vitro* and *in vivo* [41], and cells appear to have flexibility in surviving in a polyamine-deficient environment.

## Methods

### Cell culture and drug treatments

Cells were cultured with 5% CO_2_ at 37^°^C in Dulbecco’s modified Eagle’s medium (DMEM; Life Technologies) supplemented with bovine serum, 10 μg/mL ciprofloxacin (Chem-Impex Int’l), 100 IU/mL penicillin (Life Technologies), and 100 μg/mL streptomycin (Life Technologies). Huh7 cells were obtained from Dr. Susan L. Uprichard and maintained in media containing 10% heat inactivated fetal bovine serum (FBS; GeminiBio). Murie embryonic fibroblasts (MEFs) from Dorothy Sojka were maintained in 10% FBS. Vero E6 cells were obtained from BEI Resources and maintained in media containing 10% heat inactivated newborn calf serum (NBCS; GeminiBio). Difluoromethylornithine (DFMO; TargetMol) and N1-Guanyl-1,7-diaminoheptane (GC7; Cayman Chemical) were dissolved in sterile water. TOFA (Cayman Chemical), C75 (Cayman Chemical), and Etomoxir (Cayman Chemical) were dissolved in dimethyl sulfoxide (DMSO). For all treatments, cells were seeded in fresh media containing 2% serum and allowed to attach overnight prior to drug treatment. For DFMO treatment, cells were incubated for 96 hours to allow for polyamine depletion. For GC7 treatment, cells were co-treated with 500 mM aminoguanidine (Sigma-Aldrich) and incubated for 24 hours. For TOFA, C75, and etomoxir treatment, cells were incubated for 24 hours.

### Immunofluorescence

Huh7 cells were seeded to coverslips and treated and infected as indicated. Cells were fixed in 0.7% paraformaldehyde and stained with antibodies against dsRNA (J2, Sigma Aldrich), BODIPY (Invitrogen), Nile red (Invitrogen), or DAPI (Vector Labs), as appropriate. Cells were imaged on DeltaVision wide field fluorescent microscope (Applied Precision, GE) equipped with a digital camera (CoolSNAP HQ; Photometrics) and a 60X objective lens. Images were processed using SoftWoRx deconvolution software (Applied Precision, GE) and analyzed by Imaris 8.4.1 (Bitplane). An algorithm was designed in Imaris to generate surfaces around the signal of interest and surface area, volume, and counts of these surfaces were measured. All images within the same experiment were analyzed in the same manner and the same algorithm was applied.

### Polyamine TLC

Confluent cells were trypsinized and centrifuged to pellet cells. Cell pellets were washed with PBS and then resuspended in 2% perchloric acid and incubated overnight at 4^°^C. Polyamines were labeled by combining 1 volume of lysed cells with 2 volumes of 5 mg/mL dansyl chloride (Sigma-Aldrich) in acetone and 1 volume of saturated sodium bicarbonate (Sigma-Aldrich). After overnight incubation, the reaction was quenched with 1 volume of 150 mg/mL L-proline (Thermo Scientific Chemicals). Labeled polyamines were extracted with ½ volume of toluene (Sigma-Aldrich). Polyamine standards were also labeled as described above. Following centrifugation, samples and standards were spotted onto TLC plates (silica gel on TLC aluminum foils; Millipore Sigma). Plates were developed in cyclohexane:ethyl acetate (1:1) and visualized under UV exposure.

### MTT cell viability assay

Methylthialazole Tetrazolium (MTT; Cayman Chemical) was dissolved in PBS. Huh7 cells were seeded in 96-well in 2% FBS. Cells were treated with increasing doses of GC7 as described above. Media was removed. 50uL of serum free media and MTT (5mg/mL) were added to the wells. Plate was incubated for 3 hours at 37°C. MTT solvent was prepared by mixing 0.1% NP-40 and 4mM HCl in isopropanol. 150uL of MTT solvent was added to the wells. Plate was wrapped in foil and rocked for 15 minutes. Absorbance was read at OD = 590nm.

### RNA purification and cDNA synthesis

Media were cleared from cells, and Trizol reagent (Zymo Research) was added directly. Lysate was then collected, and RNA was purified through a Zymo RNA extraction kit. Purified RNA was subsequently used for cDNA synthesis using High Capacity cDNA Reverse Transcription Kits (Thermo-Fischer), according to the manufacturer’s protocol, with 10–100 ng of RNA and random hexamer primers.

### qPCR

Huh7 cells were seeded in 12-well plates in DMEM with 2% FBS. Cells were treated as indicated. Media was aspirated off cells, washed 1x with PBS, and 200 uL of Trizol was added to the cells. The RNA was extracted with the Zymo RNA extraction kit, converted to cDNA, and quantified by realtime PCR with SYBR Green (DotScientific) using the one-step protocol QuantStudio 3 (ThermoFisher Scientific). Relative expression was calculated using the ΔΔCT method, normalized to the β-actin qRT-PCR control, and calculated as the fraction of the untreated samples. Primers were verified for linearity using 8-fold serial diluted cDNA and checked for specificity via melt curve analysis. The primer sequences are as follows: ODC1 AGG-CCG-ACG-ATC-TAC-TAT-GT; GGC-ATC-CTG-TTC-CTC-TAC-TTC; ACLY TGC-ACT-GGA-AGT-AGA-GAA-GAT-TAC, CGA-GTA-AAG-GAC-CCA-CAG-TTT; ACC1 GCA-GGT-CAC-ACG-TCT-CTT-TAT, CCA-GCC-TGT-CAT-CCT-CAA-TAT-C; FASN CTA-GGT-TTG-ATG-CCT-CCT-TCT-T, GAT-GGC-TTC-ATA-GGT-GAC-TTC-C; CPT1A CTG-CCT-TTA-CGT-GGT-GTC-TAA, GGA-CAC-GTA-CTC-TGG-GTT-ATT-C; SREBF1 GAG-CCA-TGG-ATT-GCA-CTT-TC, AGC-ATA-GGG-TGG-GTC-AAA-TAG; Actin CAC-TCT-TCC-AGC-CTT-CCT-TC, GTA-CAG-GTC-TTT-GCG-GAT-GT.

### Lipase assay

Huh7 cells were seeded in a 96-well plate in 2% FBS (GeminiBio) containing DMEM (Sigma-Aldrich) with 5% CO_2_ at 37°C. Cells were treated with Difluoromethylornithine (DFMO; TargetMol) at 500 uM, 1 mM, and 5 mM for 4 days. The Lipase Activity Assay Kit (CaymenChemical; item700640) manual was followed by the manufacturer. Samples were prepared based on the cell lysate procedure. The cell pellet was lysed during sonication using 1X Assay buffer. Samples were spun, and supernatant was transferred to fresh tubes. Supernatant was transferred to a solid white 96-well plate, and kit master mix containing lipase substrate and thiol detector was added to each sample well. Control wells were plated according to kit manual. Fluorescence was read using an excitation wavelength of 385 nm and an emission wavelength of 515 nm. Lipase activity was calculated according to the kit manual.

### Lipid uptake assay

Cells were plated, treated with DMFO, similarly to the Lipase Assay method. Cells were washed with PBS and treated with 5% BODIPY 493/503 (Invitrogen) for 30 minutes at 37°C. Cells were washed with PBS and replenished using phenol red-free 2% FBS DMEM (ThermoFisher). After 24 hours, the supernatant was transferred to a 96-well plate and fluorescence was recorded. PBS was added to the cell wells, and the fluorescence was taken. The supernatant and cell sample fluorescence was taken at 485 nm/525 nm.

### siRNA knockdowns

Silencer Select siRNAs against ODC1 (s9821) or DHPS (s91) were manufactured by Thermo Fisher. For all knockdown experiments, Silencer Select negative control No.1 siRNA (Cat# 4390843), GAPDH positive control siRNA (Cat# 4390849) or gene-of-interest siRNAs were transfected into Huh7 cells. First, Huh7 cells were seeded to 40% confluence in culture plates one day prior to siRNA transfection. For each 6-well, 30 pmol siRNA diluted in Opti-MEM (Gibco; Cat# 31985070) was combined with 9 μL of Lipofectamine 2000 (Invitrogen, Cat# 11668019) diluted in Opti-MEM and allowed to form complexes for 15 minutes at room temperature. For each 12-well, 10 pmol siRNA diluted in Opti-MEM was combined with 1 μL of Lipofectamine 2000 diluted in Opti-MEM and allowed to form complexes for 15 minutes at room temperature. Following this short incubation, the transfection mixture was added dropwise to cells. Transfected cells were incubated at 37^°^C for 16 hours prior to a media change. Successful knockdown was determined by western blot or qPCR performed on samples collected 3 days post transfection.

### Western blots

Samples were collected with Bolt LDS Buffer and Bolt Reducing Agent (Invitrogen) and run on polyacrylamide gels. Gels were transferred using the iBlot 2 Gel Transfer Device (Invitrogen). Membranes were probed with primary antibodies for SREBF1 (ProteinTech), ACC1 (Cell Signaling), Actin (ProteinTech) and FASN (NSJ Bio) were treated with SuperSignal West Pico PLUS Chemiluminescent Substrate (ThermoFisher Scientific) and visualized on ProteinSimple FluorChem E imager.

### Electron microscopy

Huh7 cells were seeded in 6-well dishes and cultured in DMEM containing 2% FBS with 5% CO2 at 37^°^C. Drug treatments were performed as described above. Following incubation after drug treatment, cells were washed with PBS, trypsinized and centrifuged to pellet. Cells were fixed in 4% glutaraldehyde in cacodylate buffer (Electron Microscopy Sciences, Hatfield, PA) overnight at 4^°^C. Fixed cellular samples were sectioned into ultrathin sections cut at -120^°^C with a cryo-diamond knife. Sections were transferred to a Formvar/carbon-coated 200-mesh copper grid (Electron Microscopy Sciences) held in place by negative-action tweezers. After five minutes, the edge of the grid was blotted with a wedge of Whatman filter paper to remove excess liquid. The cells were negatively stained by immersing the grid in 1% solution of phosphotungstic acid (PTA) (pH 6.8) for 1 minute. Following negative staining with 1% PTA solution, the edge of the grid was blotted with a wedge of Whatman filter paper and the sample was allowed to dry for 5 minutes. The sample was placed into a grid storage box and dried for 24 hours prior to imaging with a Philips CM120 transmission electron microscope (TSS Microscopy, Beaverton, OR) equipped with a BioSprint 16 megapixel digital camera (Advanced Microscopy Techniques, Woburn, MA).

### Seahorse

Huh7 cells were seeded in a 96-well Seahorse XF Cell Culture Microplate and cultured in DMEM containing 2% FBS with 5% CO2 at 37^°^C (for 5 days total prior to running assay). All drug treatments were performed as described above one day after seeding cells. On the day of the assay, cell culture growth medium was changed to Seahorse XF DMEM supplemented with 1 mM pyruvate, 2 mM glutamine, and 10 mM glucose and incubated in a 37^°^C non-CO2 incubator for 1 hour prior to the assay. The sensor cartridge was loaded with 1.5 μM Oligomycin, 2 μM FCCP, and 0.5 μM Rotenone + antimycin A. Samples were then run on the Agilent Seahorse XF Analyzer. Data were analyzed using Wave software and normalized to protein concentration in each well based on values obtained using BCA assay.

### Fatty acid oxidation analysis

Huh7 cells were seeded in a 24-well plate and cultured in DMEM supplemented with 2% FBS with 5% CO_2_ at 37^°^C. After overnight incubation, cells were treated with drugs as described above. Fatty acid oxidation assay was performed according to the manufacturer’s directions (Biomedical Research Service Center, Cat#E-141). Cells were lysed using ice-cold 1x Cell Lysis Solution and centrifuged. Cell supernatants were harvested and stored at -80^°^C until the assay was performed. On the day of the assay, control solution was prepared by diluting FAO assay solution in water and reaction solution was prepared by combining diluted FAO assay solution with FAO substrate and kept on ice. Lysates were thawed quickly and 20 μL of each sample were pipetted onto a 96-well plate in duplicate. Then 50 μL of control solution were added to one set of samples and 50 μL of reaction solution was added to the other set of samples. Samples were then incubated covered for 1 hour at 37^°^C in a non-CO2 incubator. The formation of INT-formazan was assessed by measuring absorbance at 492 nm using a SpectraMax iD3 microplate reader (Molecular Devices). Lastly, protein concentration was determined for all samples by performing Bradford protein assay (Bio-Rad, Cat# 5000002). Enzyme activity after 1 hour was calculated using the following equation: FAO activity (IU/L) = μmol/(L*min) = (ODreaction–ODcontrol) x 1000 x 70 μL/ (60 min x 0.5 cm x 18 x 20 μL) = (ODreaction–ODcontrol) x 6.48. Data were normalized by protein concentration and graphed as units/μg.

### Glycolysis analysis

Huh7 cells were seeded in a 96-well plate and cultured in DMEM supplemented with 2% FBS with 5% CO2 at 37^°^C. After overnight incubation, drug treatments were performed as described above. Lactate-Glo Assay was performed according to the manufacturer’s directions (Promega, Cat#J5021). On the day of the assay, media was collected to measure secreted lactose. Following washes, 25 μL of PBS and 12.5 μL of Inactivation Solution (0.6 N HCl) was added to the cells. Then 12.5 μL of Neutralization Solution (1M Tris base) was added and mixed by shaking the plate for 30 seconds. Lastly, 50 μL of Lactate Dehydrogenase Reagent was added and mixed by shaking the plate for 30 seconds. The plate was incubated for 60 minutes at room temperature prior to recording luminescence in a white 96-well plate on a GloMax Discover Microplate reader (Promega). For this assay, PBS only wells were negative controls and were used to measure the background signal. Positive controls were wells that contained spiked in lactate from the 10 mM lactate stock.

### Virus stock preparation

CHIKV (strain 181/25; BEI Resources) was derived from passage of virus on Vero cells. Supernatants containing progeny virus were collected, clarified by centrifugation and stored at -80^°^C. Viral titers were determined by plaque assay using Vero cells. HRV1A and HRV2 were provided by Dr. Bill Jackson and derived from passage of virus on Vero cells. The following reagents were obtained through BEI Resources, NIAID, NIH: chikungunya virus, 181/25, NR-56523; La Crosse virus, NR-540; Mayaro virus, NR-49911.

### Virus titration and infection

Virus was enumerated by plaque assay. Serial dilutions of collected supernatants were prepared in serum-free DMEM and inoculated over confluent Vero cells for 30 minutes at 37^°^C. Cells were overlaid with 0.1% agarose in DMEM supplemented with 2% NBCS. Samples were incubated for 2 days prior to fixation with 4% formalin and staining with crystal violet solution. For each sample, plaques were enumerated and calculations were performed that accounted for the dilution of the supernatant to determine the number of plaque-forming units per milliliter (PFU). Cells were infected at a multiplicity of infection (MOI) of 0.1 unless otherwise noted. Viral stocks were maintained at -80°C. Viral titers were enumerated via plaque assay.
